# Association of Brain Reward Response With Body Mass Index and Ventral Striatal-Hypothalamic Circuitry Among Young Women With Eating Disorders

**DOI:** 10.1001/jamapsychiatry.2021.1580

**Published:** 2021-06-30

**Authors:** Guido K. W. Frank, Megan E. Shott, Joel Stoddard, Skylar Swindle, Tamara L. Pryor

**Affiliations:** 1 Department of Psychiatry, University of California at San Diego, San Diego; 2 Department of Psychiatry, University of Colorado, Anschutz Medical Campus, Aurora; 3 Eating Disorder Care, Denver, Colorado

## Abstract

**Importance:**

Eating disorders are severe psychiatric disorders; however, disease models that cross subtypes and integrate behavior and neurobiologic factors are lacking.

**Objective:**

To assess brain response during unexpected receipt or omission of a salient sweet stimulus across a large sample of individuals with eating disorders and healthy controls and test for evidence of whether this brain response is associated with the ventral striatal-hypothalamic circuitry, which has been associated with food intake control, and whether salient stimulus response and eating disorder related behaviors are associated.

**Design, Setting, and Participants:**

In this cross-sectional functional brain imaging study, young adults across the eating disorder spectrum were matched with healthy controls at a university brain imaging facility and eating disorder treatment program. During a sucrose taste classic conditioning paradigm, violations of learned associations between conditioned visual and unconditioned taste stimuli evoked the dopamine-related prediction error. Dynamic effective connectivity during expected sweet taste receipt was studied to investigate hierarchical brain activation between food intake relevant brain regions. The study was conducted from June 2014 to November 2019. Data were analyzed from December 2019 to February 2020.

**Main Outcomes and Measures:**

Prediction error brain reward response across insula and striatum; dynamic effective connectivity between hypothalamus and ventral striatum; and demographic and behavior variables and their correlations with prediction error brain response and connectivity edge coefficients.

**Results:**

Of 317 female participants (197 with eating disorders and 120 healthy controls), the mean (SD) age was 23.8 (5.6) years and mean (SD) body mass index was 20.8 (5.4). Prediction error response was elevated in participants with anorexia nervosa (Wilks λ, 0.843; *P* = .001) and in participants with eating disorders inversely correlated with body mass index (left nucleus accumbens: *r* = −0.291; 95% CI, −0.413 to −0.167; *P* < .001; right dorsal anterior insula: *r* = −0.228; 95% CI, −0.366 to −0.089; *P* = .001), eating disorder inventory–3 binge eating tendency (left nucleus accumbens: *r* = −0.207; 95% CI, −0.333 to −0.073; *P* = .004; right dorsal anterior insula: *r* = −0.220; 95% CI, −0.354 to −0.073; *P* = .002), and trait anxiety (left nucleus accumbens: *r* = −0.148; 95% CI, −0.288 to −0.003; *P* = .04; right dorsal anterior insula: *r* = −0.221; 95% CI, −0.357 to −0.076; *P* = .002). Ventral striatal to hypothalamus directed connectivity was positively correlated with ventral striatal prediction error in eating disorders (*r* = 0.189; 95% CI, 0.045-0.324; *P* = .01) and negatively correlated with feeling out of control after eating (right side: *r* = –0.328; 95% CI, –0.480 to –0.164; *P* < .001; left side: *r* = –0.297; 95% CI, –0.439 to –0.142; *P* = .001).

**Conclusions and Relevance:**

The results of this cross-sectional imaging study support that body mass index modulates prediction error and food intake control circuitry in the brain. Once altered, this circuitry may reinforce eating disorder behaviors when paired with behavioral traits associated with overeating or undereating.

## Key Points

**Question:**

Is brain reward response associated with specific behaviors across the eating disorder diagnostic spectrum?

**Findings:**

In this cross-sectional functional brain imaging study of 197 women with anorexia nervosa, other specified feeding and eating disorders, bulimia nervosa, and binge eating disorder and a matched cohort of 120 healthy controls, brain salience response was significantly inversely correlated with body mass index and binge eating severity and positively correlated with ventral striatal-hypothalamic circuitry.

**Meaning:**

Results of this study suggest that eating disorder behaviors change brain reward processing, which may alter food intake control circuitry and reinforce the individual’s eating disorder behavior.

## Teaser

This cross-sectional study assesses brain response during unexpected receipt or omission of a salient sweet stimulus in women with eating disorders and healthy controls to test for evidence of whether this brain response is associated with the ventral striatal-hypothalamic circuitry.

## Introduction

Eating disorders are severe psychiatric disorders with high mortality.^1^ Anorexia nervosa (AN) is characterized by severe underweight with intermittent binge eating or purging episodes; individuals with bulimia nervosa (BN) are at normal to high weight and regularly binge and purge. Binge-eating disorder (BED) is associated with binge-eating episodes and frequently elevated body weight.^2^ Eating disorders that do not meet full criteria for those diagnoses have been recognized as specific subgroups within the other specified feeding and eating disorders (OSFED) category of the *DSM-5*. Food restriction, episodic binge eating, or purging vary across the diagnostic groups, whereas body dissatisfaction and drive for thinness are typically elevated across all eating disorders, as are anxious traits and sensitivity to salient stimuli. Identifying how those behaviors are associated with particular biologic mechanisms could help create a better understanding of the underlying eating disorder pathophysiologic factors and development of specific treatments.^3^ To adopt a dimensional conceptualization of eating disorder specific behaviors and neurobiologic factors, we recruited individuals across the eating disorder spectrum and applied the prediction error construct from the National Institute of Mental Health Research Domain Criteria (RDoC) project.^4^

Brain reward circuits have been repeatedly implicated in eating disorders, and altered reward learning may play a particularly important role.^5^ In reward learning, the difference between an expectation and outcome yields a prediction error, a dopamine-associated signal that reinforces new associations.^6,7^ The direction of the prediction error is indicated by its sign, which indicates a better (positive) or worse (negative) outcome than expected. The absolute value reflects the degree of deviation of the outcome from the expectation and is related to surprise or conceptualized as a motivational salience signal.^8,9^ The dopamine system adapts in opposite directions to extremes of food intake.^10,11,12,13^ Food restriction enhances dopamine circuit activity^14,15^ and excessive food intake downregulates dopamine circuit activity,^12^ which could be relevant for eating disorder pathophysiologic factors.^16,17,18,19^ Studies in AN found elevated prediction error response to taste and monetary stimuli compared with healthy controls but a lower response in small studies in individuals with BN and individuals with overweight.^20,21,22,23^ Those studies suggested that the prediction error signal is inversely correlated with eating disorder behaviors from restrictive to loss of control food intake (binge eating).^24^ Furthermore, prediction error response was positively correlated in adolescent AN with ventral striatum-hypothalamus directed effective connectivity, a circuitry that has been associated with food intake control.^23^

For consistency with the RDoC approach, we studied a large group of individuals with eating disorders, varying on a spectrum of restrictive undereating to loss-of-control overeating. To validate previous results, we also recruited a healthy control group. First, we hypothesized that we would find inverse correlations between prediction error response and eating disorder behavior from undereating to overeating, as reflected in body mass index (BMI) and binge eating severity. This hypothesis would support basic and translational science research by externally validating a core behavioral dimension via its associations with reward-responsiveness. Second, we hypothesized that effective connectivity would be directed from the ventral striatum to the hypothalamus in the eating disorder sample. This hypothesis would support a potential trait mechanism across eating disorders to attempt to control eating drive.^23^ Third, we hypothesized that associations between biological and behavioral data may help develop a model to explain how traits, eating disorder behaviors, and neurobiologic factors interact and reinforce the often chronic nature of eating disorders.^25^

## Methods

### Participants

The Colorado Multiple institutional review board approved the study. All participants provided written informed consent. We recruited 197 women with an eating disorder: 69 AN restricting subtype, 22 AN binge-eating/purging subtype, 17 OSFED atypical AN subtype, 17 OSFED purging disorder subtype, 56 BN, 3 OSFED binge-eating subtype, and 13 binge eating disorder (BED). Participants with eating disorders were recruited from eating disorder partial hospitalization specialty care (EDCare Denver or Children’s Hospital Colorado) within the first 2 weeks of treatment, to mitigate effects of acute starvation or dehydration.^26^ Following RDoC instructions, we recruited any interested patient with eating disorders who was admitted to treatment. In addition, we recruited 120 women as healthy controls (HCs) through local advertisements. The study was conducted from June 2014 to November 2019. Data were analyzed from December 2019 to February 2020. This study followed the Strengthening the Reporting of Observational Studies in Epidemiology (STROBE) reporting guideline for cross-sectional studies.

Participants were right-handed without history of head trauma, neurological disease, major medical illness, bipolar disorder, psychosis, or current (past 3 months) substance use disorder. The healthy controls were studied during the first 10 days of the menstrual cycle to reduce potential hormonal variations. For eating disorders, treatment stage was the primary variable we controlled for, but we recorded days from last menstrual cycle as a proxy to test for hormonal variation.

### Assessments

Psychiatric diagnoses were assessed using the Structured Clinical Interview for *DSM-5* by a doctoral-level interviewer.^27^ Participants completed the Eating Disorder Inventory–3 (EDI-3) for drive for thinness (intense fear of weight gain), bulimia (tendency to engage in binge eating), body dissatisfaction (discontentment with size of body regions),^28^ Revised Sensitivity to Punishment and Reward Questionnaire,^29^ State-Trait Anxiety Inventory,^30^ Temperament and Character Inventory for Novelty Seeking and Harm Avoidance,^31^ and Beck Depression Inventory-II^32^; participants blindly rated sugar solutions for sweetness and pleasantness using a 9-point Likert scale. A subset of participants (eating disorder, n = 128, HC, n = 84) completed the Eating Expectancy Inventory for eating leads to feeling out of control.^33^

### Brain Imaging Methods

#### Functional Magnetic Resonance Imaging

Between 7:00 am and 9:00 am on the study day, participants with eating disorders ate their meal-plan breakfast and HC ate a quality-matched and calorie-matched breakfast (Table 1). Brain imaging was performed between 8:00 am and 9:00 am using the 3-T Signa (General Electric Company) or Skyra 3-T scanner (Siemens) (eMethods 1 in the Supplement). A scanner covariate was included in the multivariate analysis of covariance model for imaging group contrasts.

**Table 1.** Participant Demographic and Behavioral Characteristics

Variable	Mean (SD)					MANOVA analysis			
	HC (n = 120)	AN (n = 91)	OSFEDr (n = 34)	BN (n = 56)	BED (n = 16)	Partial η2	Statistic	*P* value	Post hoc differences
Age, y	25.15 (4.95)	21.85 (5.82)	22.10 (5.92)	23.52 (4.65)	28.60 (7.39)	0.104	*F* = 9.1	<.001	HC, BED>AN (*P* < .001); HC>OSFEDr (*P* < .05); BED>OSFEDr (*P* < .001); BED>BN (*P* < .05)
BMI	21.49 (1.64)	16.39 (1.05)	20.66 (3.09)	23.21 (7.06)	32.92 (9.52)	0.672	*F* = 159.5	<.001	HC, OSFEDr, BN, BED>AN (*P* < .001); BN>OSFEDr (*P* < .01); BED>HC, OSFEDr, BN (*P* < .001)
High lifetime BMI	22.40 (1.92)	20.83 (2.27)	25.27 (5.35)	27.32 (8.56)	34.84 (10.59)	0.362	*F* = 41.6	<.001	HC, OSFEDr, BN, BED>AN (*P* < .001); BED>HC, OSFEDr (*P* < .001); BED>BN (*P* < .01); BN>HC (*P* < .001)
Low lifetime BMI	19.94 (1.54)	14.78 (1.62)	17.49 (3.37)	18.75 (4.22)	24.05 (6.82)	0.517	*F* = 77.7	<.001	HC, OSFEDr, BN, BED>AN (*P* < .001); HC, BED>OSFEDr, BN (*P* < .001); BN, BED>HC (*P* < .001)
Novelty seeking^a^	18.91 (5.54)	15.52 (6.53)	16.53 (5.75)	18.89 (6.81)	20.75 (3.61)	0.076	*F* = 6.4	<.001	HC>AN (*P* < .001); BN>AN (*P* < .01); BED>AN (*P* < .05)
Harm avoidance^a^	10.74 (5.23)	21.85 (7.63)	23.94 (6.86)	23.96 (6.49)	17.56 (7.23)	0.402	*F* = 52.5	<.001	AN, OSFEDr, BN>HC (*P* < .001); BED>HC (*P* < .01); OSFEDr, BN>BED (*P* < .05)
Depression^b^	1.64 (2.07)	27.65 (11.81)	32.27 (11.90)	28.75 (11.36)	17.00 (12.33)	0.634	*F* = 128.2	<.001	AN, OSFEDr, BN, BED>HC (*P* < .001); AN, BN>BED (*P* < .01); OSFEDr>BED (*P* < .001)
Drive for thinness^c^	1.98 (2.96)	19.07 (7.44)	21.73 (6.77)	22.05 (5.38)	18.44 (6.74)	0.611	*F* = 121.6	<.001	AN, OSFEDr, BN, BED>HC (*P* < .001)
Bulimia^c^	0.76 (1.03)	5.59 (7.09)	7.18 (6.91)	18.18 (7.68)	19.44 (7.33)	0.548	*F* = 93.9	<.001	AN, OSFEDr, BN, BED>HC (*P* < .001); BN, BED>AN, OSFEDr (*P* < .001)
Body dissatisfaction^c^	4.22 (4.96)	24.72 (10.27)	31.61 (9.03)	29.98 (9.04)	27.50 (7.91)	0.602	*F* = 116.3	<.001	AN, OSFEDr, BN, BED>HC (*P* < .001); OSFEDr>AN (*P* < .001); BN>AN (*P* < .05)
Intolerance of uncertainty^d^	48.92 (11.78)	83.76 (19.87)	82.79 (24.39)	87.66 (22.13)	72.75 (20.99)	0.427	*F* = 58.0	<.001	AN, OSFEDr, BN, BED>HC (*P* < .001)
Reward sensitivity^e^	5.07 (3.35)	7.19 (4.02)	7.48 (2.51)	8.11 (3.52)	8.50 (3.27)	0.129	*F* = 11.5	<.001	AN, BN>HC (*P* < .001); OSFEDr, BED>HC (*P* < .01)
Punishment sensitivity^e^	4.75 (3.29)	12.21 (4.41)	13.03 (4.43)	13.32 (3.70)	10.31 (4.22)	0.437	*F* = 60.4	<.001	AN, OSFEDr, BN, BED>HC (*P* < .001)
State anxiety^f^	25.95 (6.35)	55.16 (12.35)	58.38 (12.75)	55.46 (13.08)	42.20 (14.83)	0.565	*F* = 100.9	<.001	AN, OSFEDr, BN, BED>HC (*P* < .001); AN, BN>BED (*P* < .05); OSFEDr>BED (*P* < .01)
Trait anxiety^f^	27.35 (5.65)	56.20 (11.97)	59.29 (11.26)	61.05 (11.27)	46.31 (13.80)	0.576	*F* = 105.4	<.001	AN, OSFEDr, BN, BED>HC (*P* < .001); OSFEDr>BED (*P* < .05); BN>BED (*P* < .001)
Eating leads to feeling out of control^g^	5.19 (2.41)	19.14 (5.79)	22.30 (5.20)	24.05 (4.19)	23.14 (3.89)	0.689	*F* = 114.8	<.001	AN, OSFEDr, BN, BED>HC (*P* < .001); OSFEDr>AN (*P* < .05); BN>AN (*P* < .001)
Sucrose pleasantness	5.03 (2.27)	4.23 (2.44)	3.50 (2.14)	4.54 (2.60)	5.44 (2.61)	0.046	*F* = 4.2	.003	HC>OSFEDr (*P* < .01); BED>OSFEDr (*P* < .05)
Sucrose sweetness	7.98 (0.87)	8.19 (0.98)	7.56 (1.46)	8.29 (0.89)	8.19 (0.98)	0.044	*F* = 3.2	.01	AN, BN>OSFEDr (*P* < .05)
Binge frequency (weekly)	0	1.80 (7.26)	0.25 (1.09)	14.50 (15.69)	4.38 (2.51)	0.615	*F* = 96.5	<.001	BN>HC, AN, OSFEDr, BED (*P* < .001)
Purge frequency (weekly)	0	3.05 (10.22)	3.67 (6.28)	16.02 (17.27)	0	0.426	*F* = 43.4	<.001	BN>HC, AN, OSFEDr, BED (*P* < .001)
Breakfast calories, kcal	605 (133)	584 (153)	605 (180)	596 (185)	623 (112)	0.005	*F* = 0.4	.80	NA
Antidepressant use, No. (%)	0	44 (48.4)	24 (70.6)	33 (58.9)	7 (43.8)	NA	χ^2^ = 6.1	.10	NA
Antipsychotic use, No. (%)	0	14 (15.4)	6 (17.6)	7 (12.5)	0	NA	χ^2^ = 3.3	.35	NA
MDD, No. (%)	0	43 (47.3)	20 (58.8)	31 (55.4)	4 (25)	NA	χ^2^ = 6.0	.11	NA
OCD, No. (%)	0	10 (11.0)	8 (23.5)	8 (14.3)	2 (12.5)	NA	χ^2^ = 3.2	.36	NA
PTSD, No. (%)	0	17 (18.7)	12 (35.3)	19 (33.9)	4 (25)	NA	χ^2^ = 5.8	.12	NA
Anxiety disorder, No. (%)	0	59 (64.8)	21 (61.8)	42 (75.0)	6 (37.5)	NA	χ^2^ = 7.9	.047	NA

Abbreviations: AN, anorexia nervosa, restricting subtype and binge/purge subtype; BED, binge eating disorder and other specified eating disorder, binge eating subtype; BMI, body mass index (calculated as weight in kilograms divided by height in meters squared); BN, bulimia nervosa; HC, healthy control; MANOVA, multivariate analysis of variance; NA, not applicable; OCD, obsessive-compulsive disorder; OSFEDr, other specified eating disorder restricting subtypes; PTSD, posttraumatic stress disorder.

^a^ Temperament and Character Inventory.

^b^ Beck Depression Inventory 2.

^c^ Eating Disorder Inventory-3.

^d^ Intolerance of Uncertainty Scale.

^e^ Sensitivity to Punishment and Sensitivity to Reward Questionnaire.

^f^ State-Trait Anxiety Inventory.

^g^ Eating Expectancy Inventory (a subset of participants completed the EEI: HC, n = 84; AN, n = 49; OSFEDr, n = 33; BN, n = 39; BED, n = 7).

#### Taste Reward Task

The design of this study was adapted from O’Doherty et al^34^ (eMethods 2 and eFigure 2 in the Supplement). Participants learned to associate 3 unconditioned taste stimuli (1 molar sucrose solution, no solution, or artificial saliva) with paired conditioned visual stimuli. Each conditioned visual stimulus was probabilistically associated with its unconditioned taste stimulus such that 20% of sucrose and no solution conditioned visual stimuli trials were unexpectedly followed by no solution or sucrose unconditioned taste stimuli, respectively.

#### Functional Magnetic Resonance Imaging Analysis

Image preprocessing and analysis were performed using Statistical Parametric Mapping, version 12^35^ (Wellcome Trust Centre for Neuroimaging). Images were realigned to the first volume, normalized to the Montreal Neurological Institute template, smoothed at 6-mm full width at half maximum gaussian kernel. Data were preprocessed with slice time correction and modeled with a hemodynamic response convolved function using the general linear model, including temporal and dispersion derivatives. A 128-second high-pass filter (removing low-frequency blood oxygen level dependent signal fluctuations), motion parameters (as first-level analysis regressors), and the SPM FAST (prewhitening attenuation of autocorrelation effects) were applied.^36^

#### Prediction Error Analysis

Each participant’s prediction error signal was modeled based on trial sequence (absolute of positive and negative prediction error) and regressed with brain activation across all trials^20,21,34^ (eMethods 3 in the Supplement). We extracted mean parameter estimates across all voxels from 5 predefined anatomical regions of interest (ROIs) bilaterally, based on ROIs that differentiated groups previously^23^: bilateral dorsal anterior insula (automated anatomical labeling Atlas^37^), ventral anterior insula,^37^ caudate head,^37^ ventral striatum,^38^ and nucleus accumbens.^39^

#### Effective Connectivity Analysis

We extracted ROI functional activation for trials of expected receipt of 1 molar sucrose solution (n = 80).^40^ The Tetrad-V^41^ was used to infer effective connectivity with independent multisample greedy equivalence search and linear non-gaussian orientation, fixed structure search algorithms. We extracted edge coefficients for ventral striatum-hypothalamus (hypothalamus ROI, SPM12 WFU_PickAtlas extension^42^) connectivity to test for correlations with behavior or PE values based on our previous studies^23^ (eMethods 4 in the Supplement).

### Statistical Analysis

Statistical analysis was performed with SPSS 27 software (IBM). Data were tested for normality with Shapiro-Wilk test and ranked and normalized using Rankit procedure if nonnormally distributed.^43^ Demographic and behavior data were analyzed using analysis of variance, and post hoc analyses were Bonferroni corrected. Multivariate analysis of covariance and correlation analyses were used to test effect sizes of potential confounding categorical or continuous variables such as comorbidity, medication use, or age. Variables associated with the primary outcome variable brain response were included in a group-comparison multivariate analysis of covariance and estimated marginal means post hoc comparisons Bonferroni corrected. Partial η2 was calculated for effect size in addition to power calculations. Pearson correlation analysis was used to test associations between behavior and brain activation, CIs were calculated using bootstrap (1000 samples) and results were multiple comparisons controlled using false discovery rate.^44^ All *P* values were 2-tailed, and a *P* value less than .05 was considered statistically significant.

## Results

### Demographic and Behavioral Variables

Of 317 female participants (197 with eating disorders and 120 healthy controls), the mean (SD) age was 23.8 (5.6) years and mean (SD) BMI (calculated as weight in kilograms divided by height in meters squared) was 20.8 (5.4). eTable 1 in the Supplement provides demographic and behavioral data for all groups. To increase power for comparison with HC, we combined restrictive and binge-eating/purging AN subgroups (AN, severe food restriction), OSFED atypical AN and purging disorder subgroups (OSFEDr, intermediate restrictive eating, normal BMI), and OSFED binge-eating and BED groups (BED, loss of control eating, elevated BMI). Combined subgroups were similar in BMI and psychological measures (Table 1; eFigure 1 in the Supplement). The overall age range was narrow across groups, but significantly lower in AN and OSFEDr and higher in BED compared with HC. BMI was lower in AN compared with the remaining groups and higher in BED compared with AN, OSFEDr and BN. High and low lifetime BMI showed similar patterns. Regular menses occurred in 16 participants with AN (18%, mean [SD] 15 [7] days from last cycle), 17 with OSFEDr (50%, mean [SD] 16 [8] days), all with HC (mean [SD] 6 [3] days), 33 with BN (59%, mean [SD] 12 [8] days), and 6 with BED (38%, mean [SD] 10 [6] days). Novelty seeking was lower in AN vs HC, BN and BED; harm avoidance, depression, drive for thinness, body dissatisfaction, bulimia, eating leads to feeling out of control, intolerance of uncertainty, reward and punishment sensitivity, and state and trait anxiety were higher in eating disorder groups vs HC. Sucrose pleasantness was lower in OSFEDr vs HC and AN. Frequency of weekly binge eating and purging episodes was higher in BN vs remaining groups. Breakfast calories were similar across groups.

Correlations between behavior data were consistent with previous research (eTable 2 in the Supplement). EDI-3 body dissatisfaction and EDI-3 drive for thinness were significantly positively correlated with scores for harm avoidance (body dissatisfaction *r* = 0.345; 95% CI, 0.222-0.456; *P* < .001; drive for thinness *r* = 0.357; 95% CI, 0.210-0.482; *P* < .001), depression (body dissatisfaction *r* = 0.436; 95% CI, 0.294-0.562; *P* < .001; drive for thinness *r* = 0.378; 95% CI, 0.237-0.508; *P* < .001), intolerance of uncertainty (body dissatisfaction *r* = 0.274; 95% CI, 0.138-0.396; *P* < .001; drive for thinness r = 0.400; 95% CI, 0.274-0.507; *P* < .001), sensitivity to punishment (body dissatisfaction *r* = 0.335; 95% CI, 0.201-0.459; *P* < .001; drive for thinness *r* = 0.354; 95% CI, 0.201-.492; *P* < .001), and trait anxiety (body dissatisfaction *r* = 0.448; 95% CI, 0.322-.566; *P* < .001; drive for thinness *r* = 0.480; 95% CI, 0.333-0.598; *P* < .001). EDI-3 bulimia was significantly positively correlated with BMI (*r* = 0.516; *P* < .001).

Forty-five HC and 40 participants with eating disorders took oral contraceptives (χ^2^ = 16.329; *P* < .001). Use of antidepressant or antipsychotic medication, or comorbidity with major depression, obsessive-compulsive disorder, or posttraumatic stress disorder were not differentially distributed between eating disorder groups, but comorbid anxiety disorder was (χ^2^ = 7.935; *P* = .047).

### Brain Response–Behavior Correlations

In the HC group, correlations between age, BMI, or behavior and brain imaging values were not significant or were not found after multiple comparison correction.

The eating disorder group showed significant correlations between BMI (left nucleus accumbens: *r* = −0.291; 95% CI, −0.413 to −0.167; *P* < .001; left ventral anterior insula: *r* = −0.208; 95% CI, −0.339 to −0.070; *P* = .004), binge-eating frequency (left nucleus accumbens: *r* = −0.183; 95% CI, −0.312 to −0.055; *P* = .01; left ventral anterior insula: *r* = −0.084; 95% CI, −0.212 to −0.047; *P* = .26), EDI-3 bulimia (left nucleus accumbens: *r* = −0.207; 95% CI, −0.333 to −0.073; *P* = .004; left ventral anterior insula: *r* = −0.143; 95% CI, −0.282 to −0.010; *P* = .047), trait anxiety (left nucleus accumbens: *r* = −0.148; 95% CI, −0.288 to −0.003; *P* = .04; left ventral anterior insula: *r* = −0.166; 95% CI, −0.315 to −0.001; *P* = .02), and prediction error response (Table 2; eFigure 3 in the Supplement). In a partial correlation analysis, significant correlations between regional prediction error response and BMI were found when controlling for binge-eating frequency (left nucleus accumbens *r* = -0.192; 95% CI, −0.315 to −0.059; *P* = .01; right nucleus accumbens *r* = -0.178; 95% CI, −0.324 to −0.025; *P* = .02; left caudate head *r* = −0.237; 95% CI, −0.368 to −0.095; *P* = .001; right caudate head *r* = −0.205; 95% CI, −0.348 to −0.056 *P* = .01; left ventral striatum *r* = −0.196, 95% CI, −0.329 to −0.059; *P* = .01; right ventral striatum *r* = −0.198; 95% CI, −0.334 to −0.056; *P* = .01; left dorsal anterior insula *r* = −0.154; 95% CI, −0.277 to −0.036; *P* = .04; right dorsal anterior insula *r* = −0.153; 95% CI, −0.286 to −0.017; *P* = .04; left ventral anterior insula *r* = −0.193; 95% CI, −0.319 to −0.061; *P* = .01), or EDI-3 bulimia (left nucleus accumbens *r* = −0.213; 95% CI, −0.330 to −0.088; *P* = .003; right nucleus accumbens *r* = −0.190; 95% CI, −0.322 to −0.051; *P* = .01; left caudate head *r* = −0.200; 95% CI, −0.321 to −0.074; *P* = .005; right caudate head *r* = −0.157; 95% CI, −0.287 to −0.017; *P* = .03; left ventral striatum *r* = −0.178; 95% CI, −0.299 to −0.047; *P* = .01; right ventral striatum *r* = −0.151; 95% CI, −0.275 to −0.014; *P* = .04; left dorsal anterior insula *r* = −0.151; 95% CI, −0.264 to −0.034; *P* = .04; right dorsal anterior insula *r* = −0.144; 95% CI, −0.257 to −0.023; *P* = .045; left ventral anterior insula *r* = −0.164; 95% CI, −0.290 to −0.035; *P* = .02), although significant correlations with bulimia or binge frequency were not found after controlling for BMI. Number of days from last menstrual cycle was not significantly correlated with prediction error response in any group. Exploratory analysis of the combined sample did not improve results (eMethods 5 in the Supplement).

**Table 2.** Correlation Between Regional Prediction Error Response, BMI, Binge Frequency, and EDI-3 Bulimia Score

Region	Correlation coefficient (95% CI)							
	BMI	*P* value	Binge frequency (weekly)	*P* value	EDI-3 bulimia	*P* value	Trait anxiety	*P* value
Right dorsal anterior insula	−0.228 (−0.366 to −0.089)	.001	−0.159 (−0.286 to −0.022)	.03	−0.220 (−0.354 to −0.073)	.002	−0.221 (−0.357 to −0.076)	.002
Left dorsal anterior insula	−0.228 (−0.360 to −0.103)	.001	−0.166 (−0.290 to −0.031)	.02	−0.209 (−0.344 to −0.066)	.003	−0.241 (−0.370 to −0.095)	.001
Right ventral anterior insula	−0.129 (−0.255 to 0.014)	.07	−0.129 (−0.261 to −0.001)	.08	−0.152 (−0.289 to −0.014)	.03	−0.166 (−0.320 to −0.011)	.02
Left ventral anterior insula	−0.208 (−0.339 to −0.070)	.004	−0.084 (−0.212 to 0.047)	.26	−0.143 (−0.282 to 0.010)	.047	−0.166 (−0.315 to −0.001)	.02
Right caudate head	−0.217 (−0.361 to −0.069)	.002	−0.054 (−0.189 to 0.085)	.47	−0.172 (−0.304 to −0.032)	.02	−0.137 (−0.293 to 0.038)	.06
Left caudate head	−0.287 (−0.412 to −0.152)	<.001	−0.142 (−0.274 to −0.010)	.05	−0.233 (−0.351 to −0.121)	.001	−0.162 (−0.290 to −0.019)	.02
Right ventral striatum	−0.214 (−0.341 to −0.078)	.003	−0.057 (−0.186 to 0.063)	.44	−0.174 (−0.308 to −0.037)	.02	−0.065 (−0.229 to 0.109)	.37
Left ventral striatum	−0.222 (−0.349 to −0.088)	.002	−0.068 (−0.198 to 0.061)	.36	−0.148 (−0.283 to −0.002)	.04	−0.102 (−0.240 to 0.046)	.16
Right nucleus accumbens	−0.248 (−0.383 to −0.103)	<.001	−0.145 (−0.275 to −0.009)	.048	−0.176 (−0.296 to −0.048)	.01	−0.096 (−0.246 to 0.070)	.19
Left nucleus accumbens	−0.291 (−0.413 to −0.167)	<.001	−0.183 (−0.312 to −0.055)	.01	−0.207 (−0.333 to −0.073)	.004	−0.148 (−0.288 to −0.003)	.04

Abbreviations: BMI, body mass index (calculated as weight in kilograms divided by height in meters squared); EDI-3, Eating Disorder Inventory–3.

### Effective Connectivity

**Figure 1.**
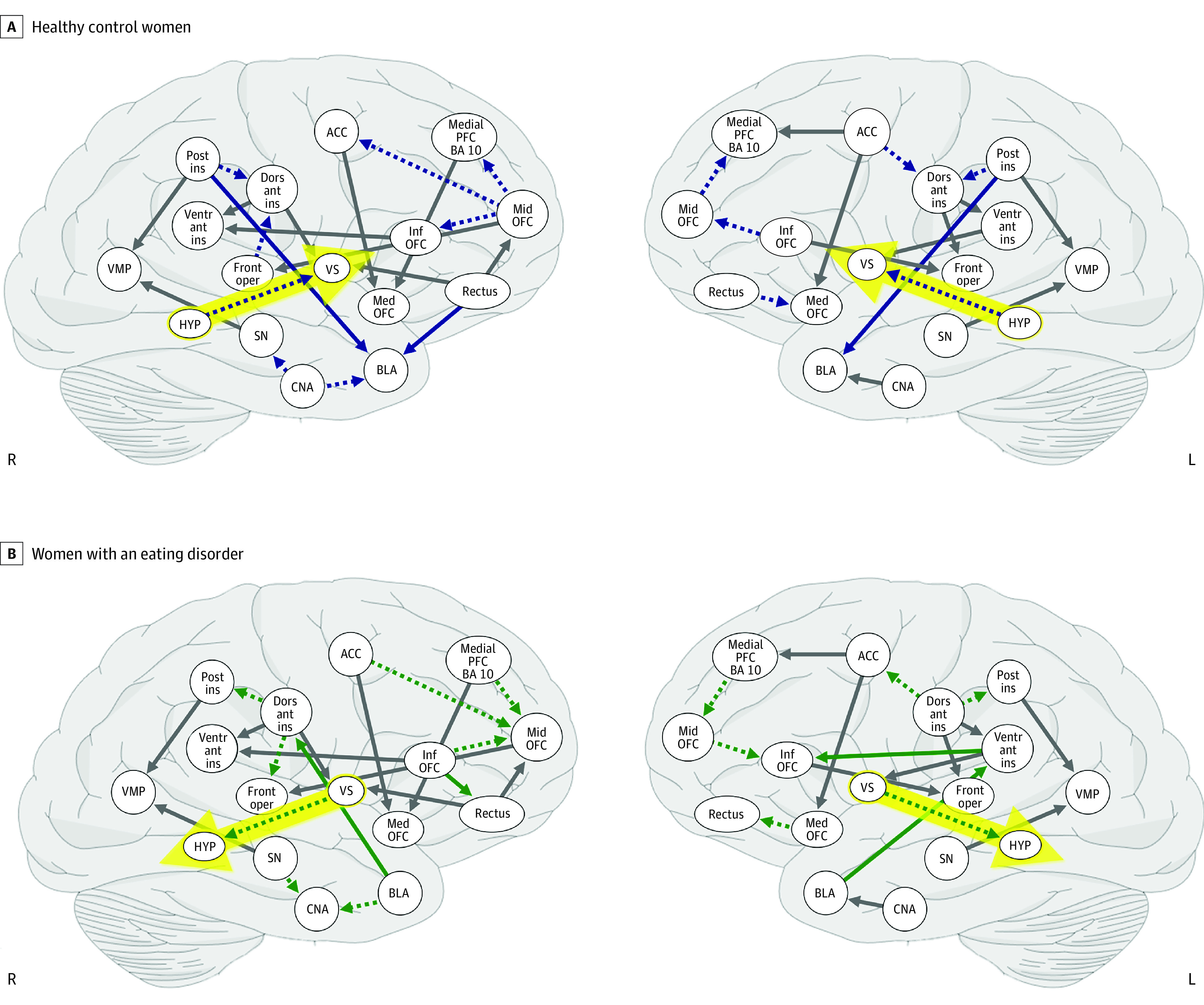
Effective Connectivity Maps Across Study Groups The yellow arrow indicates effective dynamic connectivity in opposite directions between ventral striatum and hypothalamus. ACC, anterior cingulate cortex; BA/BLA, basolateral amygdala; CAN, central nucleus of the amygdala; HYP, hypothalamus; L, left; OFC, orbitofrontal cortex; PFC, prefrontal cortex; R, right; SN, substantia nigra; VMP, ventral midbrain/pons.

Effective connectivity was directed in HC from hypothalamus to ventral striatum. In the eating disorder sample, effective connectivity was directed from ventral striatum to hypothalamus (Figure 1). eFigure 4 in the Supplement shows individual graphs for AN and BN groups.

Extracted effective connectivity edge coefficients from right ventral striatum to hypothalamus in eating disorder correlated significantly with right-sided ventral striatum prediction error response (*r* = 0.189; 95% CI, 0.045-0.324; *P* = .01); left-sided correlation was also positive but nonsignificant (*r* = 0.104; 95% CI –0.030 to 95% CI 0.231; *P* = .15). Edge coefficients correlated in eating disorders significantly in 3 ways: first, bilaterally negatively with eating leads to feeling out of control (right sided: *r* = –0.328; 95% CI, –0.480 to –0.164; *P* < .001; left sided: *r* = –0.297; 95% CI –0.439 to –0.142; *P* = .001), intolerance of uncertainty (right sided: *r* = –0.213; 95% CI –0.355 to –0.047; *P* = .004; left sided: *r* = –0.221; 95% CI, –0.354 to –0.080; *P* = .003), and sensitivity to punishment (right sided: *r* = –0.163; 95% CI, –0.295 to –0.011; *P* = .03; left sided: *r* = –0.166; 95% CI, –0.307 to –0.007; *P* = .03); second, on the right side negatively with bulimia (*r* = –0.162; 95% CI, –0.291 to –0.005; *P* = .03), and body dissatisfaction (*r* = –0.147; 95% CI, –0.279 to –0.001; *P* = .047); and third, on the left side with drive for thinness (*r* = –0.182; 95% CI, –0.317 to –0.044; *P* = .01), harm avoidance (*r* = –0.183; 95% CI, –0.334 to –0.029; *P* = .01), and trait anxiety (*r* = –0.151; 95% CI, –0.278 to –0.007; *P* = .04).

### Confounding Variables Assessment on Prediction Error Response

Multivariate analysis of covariance in the combined eating disorder group indicated no significant effect sizes for antidepressants (Wilks λ, 0.930; *P* = .21), antipsychotics (Wilks λ, 0.923; *P* = .145), major depressive disorder (Wilks λ, 0.945; *P* = .41), anxiety disorder (Wilks λ, 0.941; *P* = .09), or posttraumatic stress disorder (Wilks λ, 0.943; *P* = .38). However, there were significant effect sizes for scanner (Wilks λ, 0.843; *P* = .001), age (Wilks λ, 0.897; *P* = .03), and obsessive-compulsive disorder (Wilks λ, 0.900; *P* = .04), which were included in the prediction error group-contrast model.

### Prediction Error Group Contrasts

Prediction error response significantly differentiated groups (Wilks λ, 0.843; *P* = .001). After Bonferroni correction, prediction error remained elevated in AN compared with HC, OSFEDr and BN in the left caudate head, compared with HC and BN and BED in left nucleus accumbens, compared with HC and BN in the right nucleus accumbens, compared with HC and BED in the left ventral striatum, and compared with BN in the left dorsal insula (Table 3; eFigure 5 in the Supplement).

**Table 3.** Group Comparison for Regional Prediction Error Parameter Estimates

Region of interest	Mean (95% CI)										MANCOVA^a^				
	HC (n = 120)	SE	AN (n = 91)	SE	OSFEDr (n = 34)	SE	BN (n = 56)	SE	BED (n = 16)	SE	Partial η2	Power	*F*	*P* value	Post hoc differences
Right dorsal anterior insula	−0.096 (−0.237 to 0.055)	0.078	0.298 (0.076 to 0.529)	0.116	0.006 (−0.310 to 0.346)	0.166	−0.181 (−0.435 to 0.084)	0.130	−0.352 (−0.837 to 0.154)	0.248	0.029	0.675	2.3	.06	NA
Left dorsal anterior insula	−0.121 (−0.280 to 0.040)	0.082	0.333 (0.113 to 0.566)	0.115	−0.068 (−0.414 to 0.291)	0.176	−0.197 (−0.431 to 0.054)	0.123	−0.156 (−0.527 to 0.265)	0.200	0.036	0.771	2.8	.02	AN>BN (*P* < .05)
Right ventral anterior insula	−0.059 (−0.212 to 0.111)	0.081	0.186 (−0.044 to 0.418)	0.116	0.062 (−0.248 to 0.399)	0.166	−0.144 (−0.416 to 0.127)	0.134	−0.241 (−0.683 to 0.205)	0.224	0.017	0.412	1.3	.26	NA
Left ventral anterior insula	−0.046 (−0.204 to 0.116)	0.083	0.211 (−0.017 to 0.441)	0.118	−0.054 (−0.400 to 0.303)	0.173	−0.101 (−0.345 to 0.133)	0.119	−0.389 (−0.912 to 0.098)	0.250	0.018	0.432	1.4	.24	NA
Right caudate head	−0.145 (−0.306 to 0.015)	0.080	0.304 (0.092 to 0.514)	0.107	−0.080 (−0.431 to 0.267)	0.178	−0.034 (−0.310 to 0.246)	0.140	−0.350 (−0.836 to 0.131)	0.247	0.051	0.917	4.1	.003	AN>HC (*P* < .01); AN>OSFEDr (*P* < .05)
Left caudate head	−0.144 (−0.294 to 0.018)	0.081	0.400 (0.202 to 0.606)	0.103	−0.113 (−0.440 to 0.238)	0.176	−0.168 (−0.444 to 0.113)	0.138	−0.371 (−0.906 to 0.101)	0.256	0.069	0.980	5.7	<.001	AN>HC (*P* < .001); AN>BN (*P* < .01); AN>OSFEDr (*P* < .05)
Right ventral striatum	−0.106 (−0.248 to 0.043)	0.078	0.275 (0.057 to 0.490)	0.110	0.074 (−0.305 to 0.435)	0.192	−0.081 (−0.355 to 0.196)	0.135	−0.644 (−1.148 to −0.221)	0.244	0.039	0.818	3.2	.01	NA
Left ventral striatum	−0.110 (−0.281 to 0.046)	0.086	0.298 (0.072 to 0.515)	0.110	−0.087 (−0.441 to 0.287)	0.197	−0.012 (−0.244 to 0.228)	0.116	−0.642 (−1.061 to −0.202)	0.223	0.046	0.883	3.7	.01	AN>HC, BED (*P* < .05)
Right nucleus accumbens	−0.099 (−0.259 to 0.046)	0.079	0.326 (0.125 to 0.521)	0.103	0.089 (−0.262 to 0.473)	0.184	−0.262 (−0.533 to −0.004)	0.130	−0.380 (−0.901 to 0.155)	0.267	0.061	0.961	5.0	.001	AN>HC (*P* < .01); AN>BN (*P* < .001)
Left nucleus accumbens	−0.092 (−0.257 to 0.077)	0.087	0.336 (0.127 to 0.540)	0.103	−0.045 (−0.374 to 0.315)	0.183	−0.182 (−0.430 to 0.037)	0.114	−0.488 (−0.997 to 0.033)	0.257	0.052	0.924	4.2	.002	AN>HC, BN, BED (*P* < .05)

Abbreviations: AN, anorexia nervosa; BED, binge-eating disorder; BN, bulimia nervosa; HC, healthy controls; MANCOVA, multivariate analysis of covariance; OSFEDr, other specified eating disorder restricting subtypes.

^a^ All values were normalized; the MANCOVA model included obsessive compulsive disorder, scanner and age as factors or covariate. Post hoc group comparisons were Bonferroni corrected.

## Discussion

This cross-sectional study in a large sample of women across the eating disorder diagnostic spectrum indicates elevated prediction error response in AN compared with HC, BN, and BED, which is consistent with previous studies. In eating disorders, prediction error response was inversely correlated with BMI and binge eating behaviors. Furthermore, ventral striatal prediction error response correlated with effective connectivity from the ventral striatum to the hypothalamus in eating disorders, indicating an association between prediction error responsiveness and strength of a circuitry that has been associated with food intake control.^45^

The results support basic science studies showing that prediction error response adapts to patterns of food intake.^10,11,12,13^ Regional prediction error response was higher the more restrictive a person’s food intake was, reflected by BMI. Binge eating frequency and the EDI-3 bulimia score were also inversely correlated but not after controlling for BMI.

Previously, prediction error was associated with harm avoidance in adolescent AN, which we did not find in adults across the eating disorder spectrum.^23^ It is possible that at an earlier age prediction error response affects anxiety and triggers eating disorder behaviors.^46^ However, during longer illness, such associations may be attenuated, after eating disorder behaviors have transformed to become a way of maintaining a sense of control.^47^

Ventral striatal-hypothalamic effective connectivity during sugar tasting in opposite directions between HC and eating disorder groups, together with positive correlation with ventral striatum prediction error response, extends previous results in smaller eating disorder samples.^23,40^ Dopamine and prediction error signaling have been associated with energy homeostasis regulation,^48^ and a fear mediated dopamine circuit from the ventral striatum to the hypothalamus has been identified that inhibits food intake.^45,49,50^ A fear-driven and dopamine-mediated circuitry to suppress eating drive thus could be a trait common to all eating disorders, which is most effective in the context of sensitized dopamine circuits, reflected in high prediction error response.

**Figure 2.**
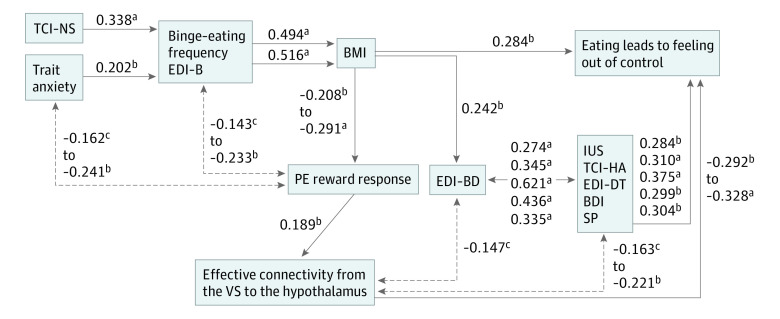
Model for Interaction Between Behaviors, Body Mass Index, and Brain Function The solid lines indicate proposed mechanistic relationships; the broken lines indicate indirect associations. Numeric values report Pearson correlation values. BDI indicates Beck Depression Inventory 2; BMI, body mass index; EDI, Eating Disorder Inventory-3; EEI, Eating Expectancy Inventory; IUS, Intolerance of Uncertainty Scale; PE, prediction error; Sensitivity to Punishment and Sensitivity to Reward Questionnaire; SP, Sensitivity to Punishment subscale; TCI indicates Temperament and Character Inventory–novelty seeking; VS, ventral striatum. ^a^*P* < .001. ^b^*P* < .01. Specific values available in eTable 2 in the Supplement. ^c^*P* < .05. Specific values available in eTable 2 in the Supplement.

The results suggest that a data-driven model of biological and behavioral interactions that promote restrictive or excessive eating behaviors is warranted (Figure 2). Consistent with previous research, trait anxiety correlated positively with the EDI-3 bulimia score, supporting that negative emotions trigger binge eating.^51^ Novelty seeking and bulimia are known risk traits for elevated BMI.^52,53^ The negative correlation between BMI and prediction error response supports basic science, indicating a modulatory effect of amount of food intake on dopaminergic circuitry.^11,12^ The positive correlation between prediction error and effective connectivity further implicates dopamine circuitry in modulating brain connectivity^54^ and suggests that higher dopaminergic activity strengthens the ventral striatal-hypothalamic food-control circuitry enabling individuals with AN to override normal hunger cues. BN or BED, however, have lower dopaminergic activity than AN, cannot maintain consistent food intake control, which facilitates intermittent binge-eating episodes.^55,56^ The negative correlation between effective connectivity and eating leads to feeling out of control could indicate that stronger food intake control circuitry leads to less of an out-of-control sensation, but this is speculative and requires further study. Behaviorally, higher BMI was associated with higher body dissatisfaction, which correlated positively with intolerance of uncertainty, harm avoidance, drive for thinness, depression and sensitivity to punishment, which also correlated positively with Trait Anxiety, consistent with previous research.^57,58,59^ Body dissatisfaction triggers drive for thinness, which reinforces and is reinforced by anxiety, depression, and punishment sensitivity, increasing poor self-esteem and further promoting eating disorder behaviors.

### Limitations

This study has limitations. The study was well powered for group comparisons, but effect sizes were small to moderate. Correlation analyses in the eating disorder sample showed moderate to large or very large effect sizes, but correlation analyses cannot prove mechanism. The prediction error model is based on dopamine function, but other neurotransmitter systems, such as serotonin, noradrenaline, or adenosine, are likely factors in reward processing and behavior control in eating disorder behaviors.^60,61,62^ Furthermore, dopamine neuronal function was not directly measured in this study and functional magnetic resonance imaging prediction error response is only an indirect approximation.^63^ Whether altered prediction error response affects food intake acutely will require further study, and inverse relationships between this brain response and BMI may also exist in other conditions. The hypothalamus ROI did not separate subnuclei. While HC were studied during the first 10 days of the menstrual cycle to keep hormonal variation low, the eating disorder population was either amenorrheic or had more days from the last menstrual cycle. Not having hormonal measures is a limitation, but days from last menstrual cycle did not correlate with brain response in either group. Because eating disorder results were either higher or lower compared with HC, we do not believe that there was a systemic confound. For the prediction error analysis, we used the unsigned (absolute) prediction error. Pleasantness ratings for the 1 molar sucrose solution varied from very high to very low. Unexpectedly receiving sucrose solution could therefore be associated with positive (better than expected) or negative (worse than expected) prediction error. Studying the absolute prediction error accounts for interindividual variation and measures degree of deviation from expectation, reducing effects of subjective pleasantness.^64,65^ Our theoretical framework was primarily based on sensitivity to salient stimuli and adaptation of the related circuitry to food intake. We believe that using the unsigned prediction error yields more reliable results, independent from individual value computation.

## Conclusions

Results of this study suggest that behavioral traits are factors in eating disorder initiation and extremes of eating and then alter prediction error–related reward response. This process reinforces in opposite ways the ventral striatal-hypothalamic food control circuitry, which is activated in response to sugar taste as a trait in eating disorders. Clinically it therefore may be important to implement weight gain in eating disorders in people with underweight and weight loss in eating disorders associated with overweight to normalize brain function and behavior. This topic is controversial, though, and the critical question remains what the best BMI for a person is in this context. Furthermore, temperamental traits are biologically oriented behaviors that affect eating disorder behaviors. Treatment modules that specifically target those behaviors may be a key element to promote behavior change and lasting recovery.

## Notes

Supplement. **eFigure 1.** Study Subject Distribution by BMI and Diagnostic Group **eMethods 1.** 3T GE Signa and Siemens Skyra 3T Scanner **eMethods 2.** Taste Reward Task Paradigm **eFigure 2.** Learned Associations and Unexpected Conditions **eMethods 3.** Temporal Difference Learning Algorithm **eMethods 4.** Effective Connectivity Analysis Methods **eTable 1.** Eight Group Comparison **eTable 2.** Correlation Analyses for Demographic and Behavior Variables **eFigure 3.** Correlation Between BMI and Left Nucleus Accumbens Prediction Error **eFigure 4.** Effective Connectivity Graphs for Anorexia and Bulimia Nervosa **eFigure 5.** Group Comparison Figure for Insula and Ventral Striatal Prediction Error Values (Normalized) **eMethods 5.** Exploratory Correlation Analysis, Combined Sample
eReferences
