# Supplementary material for: Association of Brain Reward Response With Body Mass Index and Ventral Striatal-Hypothalamic Circuitry Among Young Women With Eating Disorders
Source: JAMA Psychiatry. 2021 Jun 30;78(10):1–12. doi: 10.1001/jamapsychiatry.2021.1580 (PMC8246338; doi:10.1001/jamapsychiatry.2021.1580)
Supplement: Supplement. — eFigure 1. Study Subject Distribution by BMI and Diagnostic Group eMethods 1. 3T GE Signa and Siemens Skyra 3T Scanner eMethods 2. Taste Reward Task Paradigm eFigure 2. Learned Associations and Unexpected Conditions eMethods 3. Temporal Difference Learning Algorithm eMethods 4. Effective Connectivity Analysis Methods eTable 1. Eight Group Comparison eTable 2. Correlation Analyses for Demographic and Behavior Variables eFigure 3. Correlation Between BMI and Left Nucleus Accumbens Prediction Error eFigure 4. Effective Connectivity Graphs for Anorexia and Bulimia Nervosa eFigure 5. Group Comparison Figure for Insula and Ventral Striatal Prediction Error Values (Normalized) eMethods 5. Exploratory Correlation Analysis, Combined Sample eReferences [file jamapsychiatry-e211580-s001.pdf]

Supplemental Online Content

Frank GKW, Shott NE, Stoddard J, Swindle S, Pryor T. Association of brain reward response with body mass index and ventral striatal-hypothalamic circuitry among young women with eating disorders. *JAMA Psychiatry*. Published online June 30, 2021. doi:10.1001/jamapsychiatry.2021.1580

eFigure 1. Study Subject Distribution by BMI and Diagnostic Group
eMethods 1. 3T GE Signa and Siemens Skyra 3T Scanner
eMethods 2. Taste Reward Task Paradigm
eFigure 2. Learned Associations and Unexpected Conditions
eMethods 3. Temporal Difference Learning Algorithm
eMethods 4. Effective Connectivity Analysis Methods
eTable 1. Eight Group Comparison
eTable 2. Correlation Analyses for Demographic and Behavior Variables
eFigure 3. Correlation Between BMI and Left Nucleus Accumbens Prediction Error
eFigure 4. Effective Connectivity Graphs for Anorexia and Bulimia Nervosa
eFigure 5. Group Comparison Figure for Insula and Ventral Striatal Prediction Error Values (Normalized)
eMethods 5. Exploratory Correlation Analysis, Combined Sample
eReferences

This supplemental material has been provided by the authors to give readers additional information about their work.

© 2021 Frank GKW et al. *JAMA Psychiatry*.

eFigure 1. Study Subject Distribution by BMI and Diagnostic Group

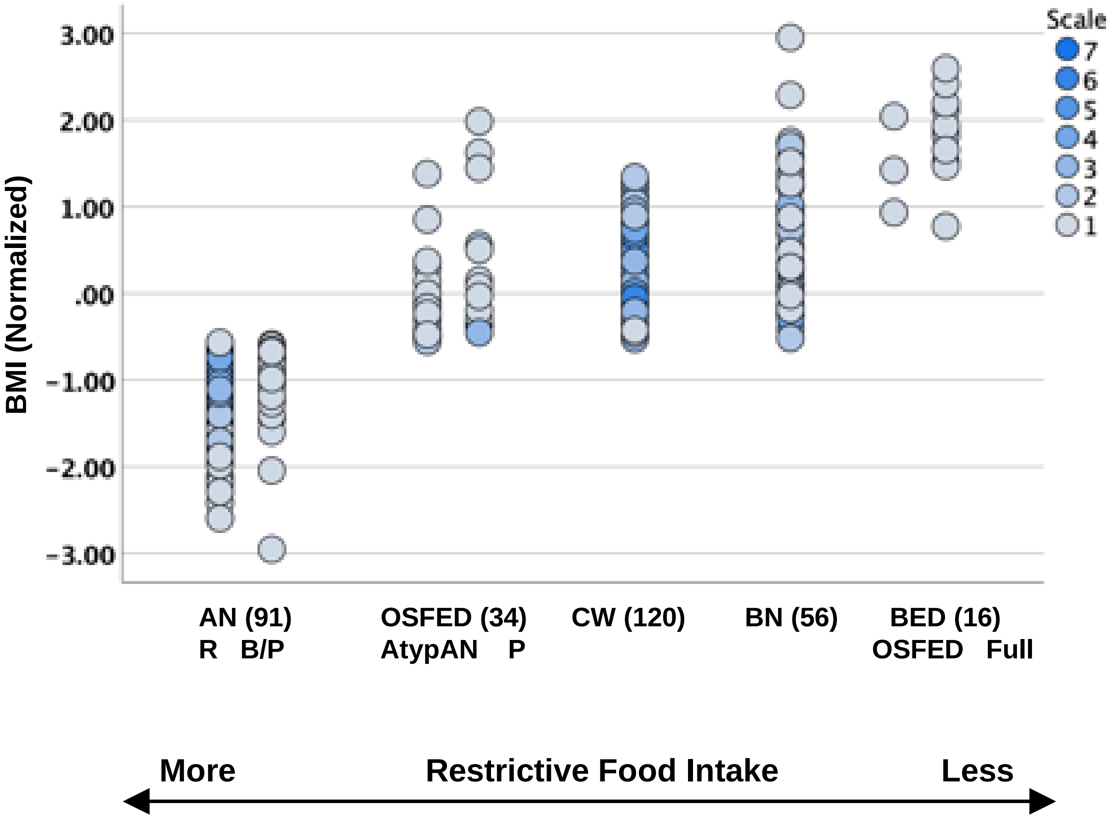

AN, anorexia nervosa; R, restricting type; B/P binge eating/purging type; OSFED, other specified eating or feeding disorder; AtypAN, atypical anorexia nervosa; P, purging disorder; CW, control women; BN, bulimia nervosa; BED, binge eating disorder.

Subject data were collected between July 2014 and December 2019.

© 2021 Frank GKW et al. *JAMA Psychiatry*.

eMethods 1. 3T GE Signa and Siemens Skyra 3T Scanner

Brain imaging was performed between 0800 and 0900 hours on a 3T GE Signa or a Siemens Skyra 3T scanner: AN (GE Signa n=52, Siemens Skyra n=39), OSFEDr (GE Signa n=10, Siemens Skyra n=24), HC (GE Signa n=54, Siemens Skyra n=66), BN (GE Signa n=23, Siemens Skyra n=33) and 10 BED (GE Signa n=10, Siemens Skyra n=6), with a three-plane scout scan (16 seconds), sagittally acquired, spoiled gradient sequence T1-weighted (172 slices, thickness=1mm, TI=450ms, TR=8ms, TE=4ms, flip angle=12°, FOV=22cm, scan matrix=64×64), and T2*-weighted echo planar scans for blood-oxygen-level-dependent (BOLD) functional activity (3.4×3.4×2.6mm voxels, TR=2100ms, TE=30ms, flip angle=70°, 28 axial slices, thickness=2.6mm, gap=1.4mm).

A Chi-square test indicated that healthy controls and individuals with eating disorders were similarly distributed across the two scanners (χ^2=0.311, p=.58). An additional MANOVA for the 10 ROIs investigated, with two factors, group (AN, OSFEDr, HC, BN, BED) and scanner, yielded a significant result for group (Wilks λ=0.812, p=.01) and scanner (Wilks λ=0.819, p<.001), but no significant group by scanner interaction (Wilks λ=0.862, p=.27). Nevertheless, to account for potential scanner effects across groups, a scanner covariate was included in the MANCOVA model for imaging group contrasts.

© 2021 Frank GKW et al. *JAMA Psychiatry*.

eMethods 2. Taste Reward Task Paradigm

The taste reward task design was adapted from O'Doherty et al.[1] Participants received three taste stimuli during fMRI imaging (28 min. total task duration): 1 molar sucrose solution (100 trials), no solution (100 trials) and artificial saliva (80 trials). Participants learned to associate each unconditioned taste stimulus (US) with a paired conditioned visual stimulus (CS) that is probabilistically associated with its US: the CS shape for sucrose was followed in 80% of trials by sucrose solution (the other 20% were followed by no solution), and the CS shape associated with no-solution was followed in 80% of the trials by no solution (the other 20% were followed by sucrose); the CS shape for artificial saliva was always followed by saliva receipt. For each subject, the first 10 trials were fixed CS shape for sucrose followed by the delivery of US sucrose to establish an initial stable association between the CS sucrose shape and US sucrose taste.[1] All other trials were fully randomized without predetermined order. Taste stimuli were applied using a customized-programmable syringe pump (J-Kem Scientific, St Louis, MO, USA) and E-Prime Software (Psychological Software Tools, Pittsburgh, PA, USA).[2] The MRI scanner radiofrequency pulse triggered taste application.[3]

Study participants were compensated for their study participation with a total of $160, prorated for completion of questionnaires, diagnostic assessment and brain imaging scan.

© 2021 Frank GKW et al. *JAMA Psychiatry*.

eFigure 2. Learned Association and Unexpected Conditions

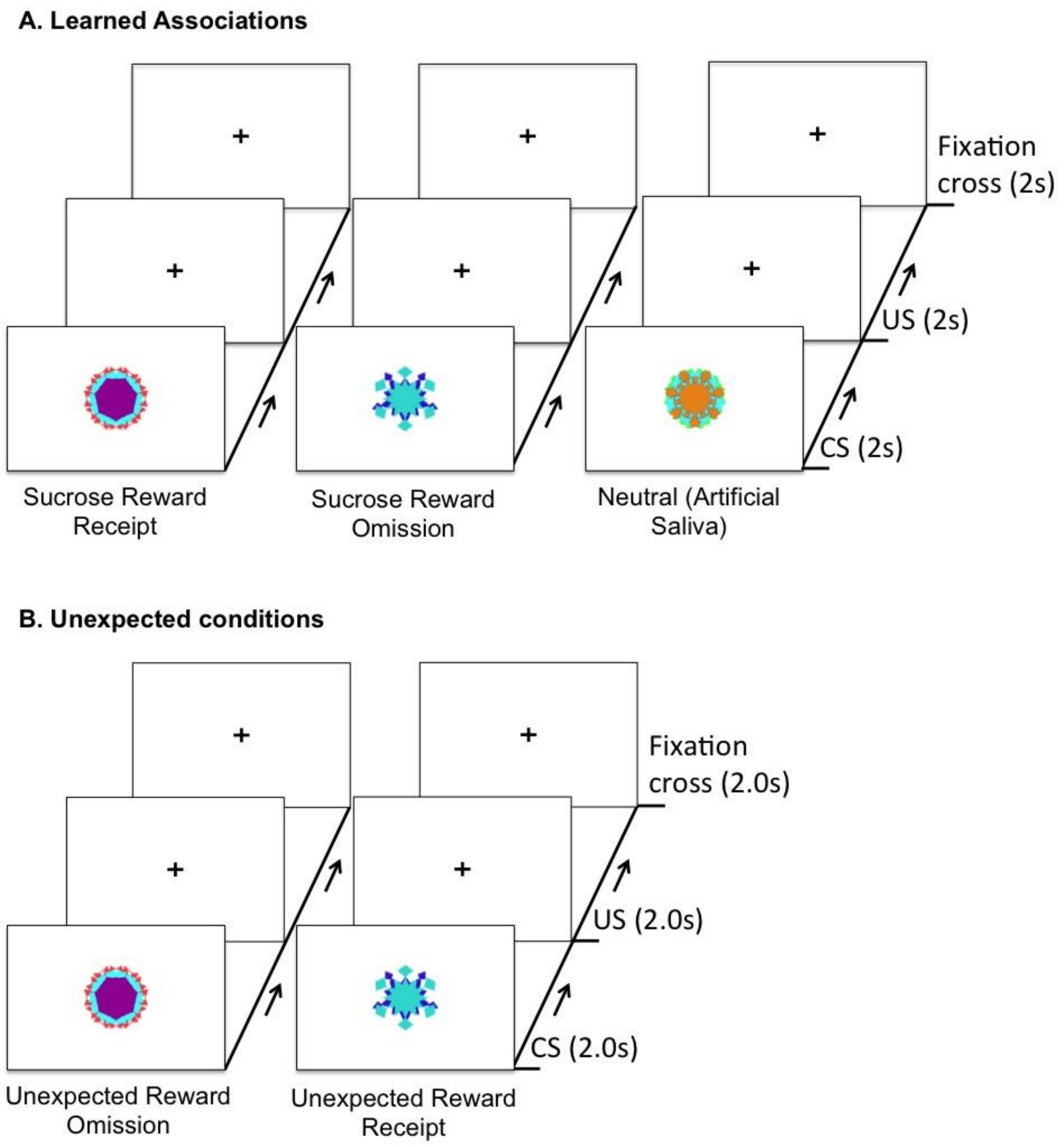

Panel A depicts the learned associations between the conditioned stimulus (CS, colored geometric shape, presented for 2 seconds (s)) and the unconditioned stimulus (US, 1ml sweet taste reward, presented for 2s). Intertrial interval was 6s. Panel B depicts the unexpected conditions where learned associations were violated during 20% of the trials.

© 2021 Frank GKW et al. *JAMA Psychiatry*.

eMethods 3. Temporal Difference Learning Algorithm

The predicted value ($\hat{V}$) at any time (*t*) within a trial is calculated as a linear product of weights (w_i) and the presence of a conditioned visual stimulus (CS) at time *t,* coded in a stimulus representation vector $x_i(t)$ where each stimulus x_i is represented separately at each moment in time:

$$V(t) = \sum_i W_i x_i(t)$$

Predicted stimulus value at time *t* is updated by comparing the predicted value at time *t*+1 to that actually observed at time *t,* leading to the prediction error $\delta(t)$:

$$\delta(t) = r(t) + \gamma\hat{V}(t+1) - \hat{V}(t)$$

where *r(t)* is the reward at time *t.* The parameter γ is a discount factor, which determines the extent to which rewards arriving sooner are more important than rewards that arrive later during the task, with γ=0.99. The weights wi relate to how likely a particular unconditioned reward stimulus (US) follows the associated CS and are updated on each trial according to the correlation between prediction error and the stimulus representation:

$$\Delta w_i = \alpha \sum_t x_i(t)\delta(t)$$

where α is a learning rate. Between slow and fast learning rates, (0.2, 0.7) a slow α=0.2 was the best fit for study groups (see below). Initial reward values were 1 for Sucrose Receipt and 0 for No Sucrose. Trial-to-trial prediction error was regressed with brain activation across all trials within each subject.

Testing of appropriateness of learning rate: Learning rates in the 0.2 ranges have been commonly used in temporal difference model studies [1,4-8] and are “thought to fall within the naturalistic range of striatal dopamine neurons[9,10]” (cited in Robinson et al.[8]). Previous studies from our group and others have suggested that the slow learning rate is the better fit.[11] Several additional tests were done in this study: For the ED group, 8 out of 10 regions, and for the HC group, 5 out of 10 regions had higher values for α=0.2 versus α=0.7. in addition, there were no

© 2021 Frank GKW et al. *JAMA Psychiatry*.

significant regional prediction error differences in either group. An additional MANCOVA comparing groups using the α=0.7 derived prediction error values showed similar group contrasts, using the same covariates.

Rationale for use of unsigned (absolute) prediction error: The prediction error calculated for each trial was modeled as an absolute (reflecting response strength) without separating positive or negative prediction error trials. This trial-to-trial calculated prediction error was then regressed with the parameter estimates derived from brain activation across all trials within each subject. The prediction error signals can be signed (positive or negative), signaling better or worse outcome than expected, or unsigned (absolute value), as the degree of deviation from expectation and related to surprise or conceptualized as a motivational salience signal.[12,13] For the prediction error analysis, we used the unsigned (absolute) prediction error. The 1M sucrose solution is uniformly rated as very sweet, while pleasantness ratings vary from very high to very low. Furthermore, even during a study sequence, the pleasantness experience can change, from initially "liking" the sweet taste, but eventually not finding it pleasant anymore. Unexpectedly receiving sucrose solution could therefore be associated with positive (better than expected) or negative (worse than expected) prediction error and we have been studying the absolute prediction error to account for inter-individual and trial-to-trial variation. We obtained post-scan pleasantness ratings for the majority of individuals in the study. Pleasantness ratings for the 1 M sucrose solution were lower post scan compared to pre-scan assessment in all groups (HC n=100, pre-scan 5.1 vs. post-scan 4.2; AN n=67, pre-scan 4.4 vs. post-scan 4.3; OSFEDr n=34, pre-scan 3.50 vs. post-scan 3.12; BN n=41, pre-scan 4.2 vs. post-scan 4.1; BED n=12, pre-scan 6.0 vs. post-scan 5.5). A paired samples t-test indicated significant differences between pre and post-scan pleasantness ratings, t=3.547, p<.001.

Using the unsigned prediction error may rather test the motivational salience aspect of this circuitry and be less related to the valuation of the stimulus.[14,15] Our theoretical framework is

© 2021 Frank GKW et al. *JAMA Psychiatry*.

primarily based on sensitivity to salient stimuli and adaptation of the related circuitry to food intake, and we believe that using the unsigned prediction error yields more reliable results, independent from individual value computation. The prediction error model is based on dopamine function but likely other neurotransmitter systems such as for instance serotonin, noradrenaline or adenosine also take part in reward processing and behavior control to drive ED behaviors.[16-18]

© 2021 Frank GKW et al. *JAMA Psychiatry*.

eMethods 4. Effective Connectivity Analysis Methods

We used the SPM12 MarsBar toolbox to extract functional time-series data for the previously examined[19] expected receipt of 1 M sucrose solution. The TETRAD V program[20] was next used to infer effective connectivity with Independent Multiplesample Greedy Equivalence Search (IMaGES) and Linear non-gaussian Orientation, Fixed Structure search algorithms. This analysis aimed to understand causal relations among neuronal populations whose activity gives rise to observed fMRI signals in spatially localized regions of interest. Results analyses are presented as directed graphs, where nodes or vertices in the graph represent brain regions and directed edges in the graph represent relatively direct causal influences of one region on another. The Independent Multiplesample Greedy Equivalence Search (IMaGES) is a modification of the Greedy Equivalence Search (GES) that allows analysis of multiple data sets. GES begins with an empty graph whose vertices are the recorded variables and proceeds to search forward, one new connection at a time, over Markov Equivalence classes of directed acyclic graphs. Each class of models with an additional edge is scored using the Bayes Information Criterion: $-2\ln(ML) + k \ln(n)$, where ML is the maximum likelihood estimate, k is the dimension of the model (the number of directed edges plus the number of variables) and n is the sample size. The algorithm searches forward from the empty graph until no improvement in the Bayes Information Criterion score is possible, and then backward, and outputs a description of a Markov Equivalence class. The algorithm requires a computation of a series of maximum likelihood estimates and is limited to cases where approximations to such estimates can be rapidly obtained. The analysis process in IMaGES and GES is nonlinear, and therefore a comparison of a parameterized output of the GES using conventional linear models for group comparison is not recommended. IMaGES was supplemented by a Linear non-gaussian Orientation, Fixed Structure algorithm postprocessor; this leads to a precision of orientations that is greater than 90% and the precision of recall greater than 80%, that is, more edges are directed than with IMaGES alone, and with no loss of accuracy.[21]

© 2021 Frank GKW et al. *JAMA Psychiatry*.

eTable 1. Eight Group Comparison

	HC (A) (n = 120)		AN-R (B) (n = 69)		AN-BP (C) (n=22)		OSFED-Atyp (D) (n=17)		OSFED-P (E) (n=17)		BN (F) (n=56)		OSFED-BED (G) (n=3)		BED (H) (n=13)		MANOVA Analysis			
	Mean	SD	Mean	SD	Mean	SD	Mean	SD	Mean	SD	Mean	SD	Mean	SD	Mean	SD	ect Size (Partial r	F	p	comparison
Age (years)	25.15	4.95	20.93	5.19	24.73	6.83	22.74	5.93	21.46	6.02	23.52	4.65	30.42	11.55	28.18	6.71	0.134	6.813	<0.001	C, F > B*; A, H > B***; A > E*; H > E**
BMI at Scan (kg/m[2])	21.49	1.64	16.30	1.03	16.68	1.09	19.89	1.89	21.44	3.85	23.21	7.06	28.48	7.15	33.94	9.93	0.676	92.093	<0.001	A, D, E, F, G, H > B, C***; A > D*; F > D**; G, H > D***; G > A, E*; H > A, E, F***
High Lifetime BMI (kg/m[2])	22.40	1.92	20.91	2.20	20.60	2.51	23.24	2.61	27.44	6.66	27.32	8.56	32.35	12.22	35.22	10.83	0.371	47.035	<0.001	D, G > B, C*; A, E, F, H > B***; A > C**; E, F, H > C***; H > D***; E > A*; F, H > A***; H>F**
Low Lifetime BMI (kg/m[2])	19.94	1.54	14.73	1.43	14.93	2.08	16.43	3.43	18.64	3.00	18.75	4.22	19.18	3.68	24.80	6.97	0.534	4.723	<0.001	A, E, F, H > B, C***; A, H > D, F***; H > A, E***
Novelty Seeking[a]	18.91	5.54	14.58	6.26	18.45	6.64	16.76	5.52	16.29	6.13	18.89	6.81	23.00	4.58	20.23	3.35	0.097	30.747	<0.001	A, F > B***; H > B*
Harm avoidance[a]	10.74	5.23	22.38	7.89	20.18	6.64	24.88	6.44	23.00	7.32	23.96	6.49	14.33	10.26	18.31	6.68	0.411	4.797	<0.001	B, C, D, E, F > A***; H > A**
Depression[b]	1.64	2.07	26.90	11.89	29.90	11.58	32.63	13.06	31.94	11.09	28.75	11.36	14.67	12.86	17.54	12.68	0.638	73.736	<0.001	B, C, D, E, F, H > A***; D > G, H*; E > G*
Drive for thinness[c]	1.98	2.96	19.07	7.65	19.05	6.85	21.31	6.92	22.12	6.83	22.05	5.38	10.67	4.93	20.23	5.85	0.615	70.155	<0.001	B, C, D ,E, F, H > A***
Bulimia[c]	0.76	1.03	2.72	3.37	15.00	7.99	5.63	6.08	8.65	7.49	18.18	7.68	12.00	6.25	21.15	6.61	0.636	76.784	<0.001	B, C, D, E, F, H > A***; G > A**; C, E, F, H > B***; C, F, H > D***; F, H > E***
Body dissatisfaction[c]	4.22	4.96	24.71	10.06	24.75	11.24	31.06	7.38	32.12	10.57	29.98	9.04	18.00	6.25	29.69	6.63	0.607	67.266	<0.001	E > B***; B, C, D, E, F, H > A***
Intolerance of uncertainty[d]	48.92	11.78	83.20	21.63	85.50	13.13	85.24	27.29	80.35	21.65	87.66	22.13	66.33	31.66	74.23	19.27	0.431	33.488	<0.001	B, C, D, E, F, H > A***
Reward sensitivity[e]	5.07	3.35	7.04	3.92	7.64	4.37	7.41	2.43	7.56	2.68	8.11	3.52	8.00	1.00	8.62	3.62	0.130	6.598	<0.001	B > A**; C, H > A*; F > A***
Punishment sensitivity[e]	4.75	3.29	12.49	4.50	11.32	4.09	12.47	5.20	13.63	3.52	13.32	3.70	8.00	6.25	10.85	3.76	0.448	35.707	<0.001	B, C, D ,E, F, H > A***
State anxiety[f]	25.95	6.35	55.13	13.30	55.27	9.02	58.82	10.56	57.94	14.95	55.46	13.08	33.50	13.44	43.54	15.07	0.567	57.478	<0.001	B, C, D ,E, F, H > A***
Trait anxiety[f]	27.35	5.65	55.34	13.10	58.86	7.01	59.41	11.95	59.18	10.88	61.05	11.27	43.00	14.73	47.08	14.09	0.577	59.890	<0.001	B, C, D ,E, F, H > A***; F > H*
Eating leads to feeling out of control[g]	5.19	2.41	19.29	6.01	18.79	5.40	22.56	5.03	22.06	5.49	24.05	4.19	19.50	4.95	24.60	2.70	0.693	65.632	<0.001	B, C, D, E, F, H > A***; G > A*; F > B***; F > C**
Sucrose pleasantness	5.03	2.27	4.09	2.50	4.71	2.24	4.18	2.01	2.82	2.10	4.54	2.60	6.00	3.61	5.31	2.50	0.059	2.972	0.005	A > E**
Sucrose sweetness	7.98	0.87	8.17	1.01	8.24	0.89	7.53	1.63	7.59	1.33	8.29	0.89	7.67	1.53	8.31	0.86	0.047	1.939	0.063	n.s.
Binge frequency (per week)	0.00	0.00	0.00	0.00	9.55	14.71	0.12	0.38	0.40	1.55	14.50	15.69	3.67	3.25	4.54	2.44	0.739	84.187	<0.001	C, F > A, B***; C > D**; C > E*
Purge frequency (per week)	0.00	0.00	0.00	0.00	16.22	18.91	0.03	0.12	10.56	6.47	16.02	17.27	0.00	0.00	0.00	0.00	0.729	77.057	<0.001	C, F > A, B, D, H***; E > B*; E > A**; F > G*
Scan breakfast calories (kcal)	604.96	132.97	589.71	154.44	565.46	151.29	630.00	185.61	579.94	175.62	596.21	185.29	569.67	124.74	634.81	110.86	0.010	0.439	0.877	n.s.
	N	%	N	%	N	%	N	%	N	%	N	%	N	%	N	%		χ2	p	
Antidepressant use	0	0	35	50.7	9	40.9	11	64.7	13	76.5	33	58.9	1	33.3	6	46.2		7.409	0.285	
Antipsychotic use	0	0	10	14.5	4	18.2	1	5.9	4	23.5	7	12.5	0	0.0	0	0.0		4.460	0.615	
Current Major Depressive Disorder	0	0	30	43.5	13	59.1	10	58.8	10	58.8	31	55.4	1	33.3	3	23.1		7.701	0.261	
Current OCD	0	0	8	11.6	2	9.1	3	17.6	5	29.4	8	14.3	0	0.0	2	15.4		4.759	0.575	
Current PTSD	0	0	8	11.6	9	40.9	8	47.1	4	23.5	19	33.9	1	33.3	3	23.1		15.757	0.015	
Current Anxiety Disorder	0	0	42	60.9	17	77.3	9	52.9	12	70.6	42	75.0	1	33.3	5	38.5		11.099	0.085	

HC: healthy control; AN-R: anorexia nervosa, restricting subtype; AN-BP: anorexia nervosa, binge/purge subtype; OSFED-Atyp: other specified eating disorder, atypical anorexia nervosa; OSFED-P: other specified eating disorder, purging disorder; BN: bulimia nervosa; OSFED-BED: other specified eating disorder, binge eating disorder; BED: binge eating disorder; BMI: body mass index; OCD: obsessive compulsive disorder; PTSD: post traumatic stress disorder; GAD: generalized anxiety disorder; n.s.: non significant. *P < 0.05, **P < 0.01, ***P < 0.001. Significance is based on the Tukey's HSD post hoc test.

[a] Temperament and Character Inventory

[b] Beck Depression Inventory 2

[c] Eating Disorder Inventory 3

[d] Intolerance of Uncertainty Scale

[e] Sensitivity to Punishment and Sensitivity to Reward Questionnaire

[f] State-Trait Anxiety Inventory

[g] Eating Expectancy Inventory; HC (n=84), AN-R (n=35), AN-BP (n=14), OSFED-Atyp (n=16), OSFED-P (n=17), BN (n=39), OSFED-BED (n=2), BED (n=5)

© 2021 Frank GKW et al. *JAMA Psychiatry*.

eTable 2. Correlation Analyses for Demographic and Behavior Variables in ED Group

Correlations

				BMI	NS	HA	BDI	EDI3-DT	EDI3-B	EDI3-BD	IUS	SP	Trait Anxiety
**BMI**	**Pearson Correlation**				.287**	-0.068	-0.079	0.115	.516**	.242**	-0.072	-0.047	-0.01
	**Sig. (2-tailed)**				<0.001	0.362	0.291	0.12	<0.001	0.001	0.331	0.523	0.894
		**95% Confidence Interval**	**Lower**		0.163	-0.201	-0.211	-0.017	0.428	0.124	-0.193	-0.176	-0.157
			**Upper**		0.422	0.077	0.054	0.252	0.603	0.362	0.072	0.1	0.145
**NS**	**Pearson Correlation**			.287**		-.318**	-0.09	-0.053	.281**	-0.059	-.266**	-.352**	-0.081
	**Sig. (2-tailed)**			<0.001		<0.001	0.227	0.476	<0.001	0.43	<0.001	<0.001	0.277
		**95% Confidence Interval**	**Lower**	0.163		-0.427	-0.21	-0.189	0.135	-0.198	-0.381	-0.468	-0.196
			**Upper**	0.422		-0.193	0.034	0.104	0.415	0.08	-0.134	-0.218	0.035
**HA**	**Pearson Correlation**			-0.068	-.318**		.553**	.357**	0.102	.345**	.656**	.762**	.589**
	**Sig. (2-tailed)**			0.362	<0.001		<0.001	<0.001	0.17	<0.001	<0.001	<0.001	<0.001
		**95% Confidence Interval**	**Lower**	-0.201	-0.427		0.437	0.21	-0.036	0.222	0.549	0.686	0.475
			**Upper**	0.077	-0.193		0.645	0.482	0.232	0.456	0.741	0.82	0.676
**BDI**	**Pearson Correlation**			-0.079	-0.09	.553**		.378**	0.111	.436**	.513**	.485**	.741**
	**Sig. (2-tailed)**			0.291	0.227	<0.001		<0.001	0.133	<0.001	<0.001	<0.001	<0.001
		**95% Confidence Interval**	**Lower**	-0.211	-0.21	0.437		0.237	-0.035	0.294	0.373	0.367	0.667
			**Upper**	0.054	0.034	0.645		0.508	0.268	0.562	0.632	0.583	0.801
**EDI3-DT**	**Pearson Correlation**			0.115	-0.053	.357**	.378**		.279**	.621**	.400**	.354**	.480**
	**Sig. (2-tailed)**			0.12	0.476	<0.001	<0.001		<0.001	<0.001	<0.001	<0.001	<0.001
		**95% Confidence Interval**	**Lower**	-0.017	-0.189	0.21	0.237		0.117	0.526	0.274	0.201	0.333
			**Upper**	0.252	0.104	0.482	0.508		0.421	0.701	0.507	0.492	0.598
**EDI3-B**	**Pearson Correlation**			.516**	.281**	0.102	0.111	.279**		.284**	.163*	0.106	.202**
	**Sig. (2-tailed)**			<0.001	<0.001	0.17	0.133	<0.001		<0.001	0.027	0.154	0.006
		**95% Confidence Interval**	**Lower**	0.428	0.135	-0.036	-0.035	0.117		0.136	0.022	-0.055	0.051
			**Upper**	0.603	0.415	0.232	0.268	0.421		0.402	0.296	0.25	0.343
**EDI3-BD**	**Pearson Correlation**			.242**	-0.059	.345**	.436**	.621**	.284**		.274**	.335**	.448**
	**Sig. (2-tailed)**			0.001	0.43	<0.001	<0.001	<0.001	<0.001		<0.001	<0.001	<0.001
		**95% Confidence Interval**	**Lower**	0.124	-0.198	0.222	0.294	0.526	0.136		0.138	0.201	0.322
			**Upper**	0.362	0.08	0.456	0.562	0.701	0.402		0.396	0.459	0.566
**IUS**	**Pearson Correlation**			-0.072	-.266**	.656**	.513**	.400**	.163*	.274**		.597**	.564**
	**Sig. (2-tailed)**			0.331	<0.001	<0.001	<0.001	<0.001	0.027	<0.001		<0.001	<0.001
		**95% Confidence Interval**	**Lower**	-0.193	-0.381	0.549	0.373	0.274	0.022	0.138		0.465	0.452
			**Upper**	0.072	-0.134	0.741	0.632	0.507	0.296	0.396		0.69	0.673
**SP**	**Pearson Correlation**			-0.047	-.352**	.762**	.485**	.354**	0.106	.335**	.597**		.477**
	**Sig. (2-tailed)**			0.523	<0.001	<0.001	<0.001	<0.001	0.154	<0.001	<0.001		<0.001
		**95% Confidence Interval**	**Lower**	-0.176	-0.468	0.686	0.367	0.201	-0.055	0.201	0.465		0.343
			**Upper**	0.1	-0.218	0.82	0.583	0.492	0.25	0.459	0.69		0.59
**Trait Anxiety**	**Pearson Correlation**			-0.01	-0.081	.589**	.741**	.480**	.202**	.448**	.564**	.477**	
	**Sig. (2-tailed)**			0.894	0.277	<0.001	<0.001	<0.001	0.006	<0.001	<0.001	<0.001	
		**95% Confidence Interval**	**Lower**	-0.157	-0.196	0.475	0.667	0.333	0.051	0.322	0.452	0.343	
			**Upper**	0.145	0.035	0.676	0.801	0.598	0.343	0.566	0.673	0.59	

**** Correlation is significant at the 0.01 level (2-tailed).**

*** Correlation is significant at the 0.05 level (2-tailed).**

Bootstrap results are based on 1000 bootstrap samples

© 2021 Frank GKW et al. *JAMA Psychiatry*.

eFigure 3. Correlation Between BMI and Left Nucleus Accumbens Prediction Error

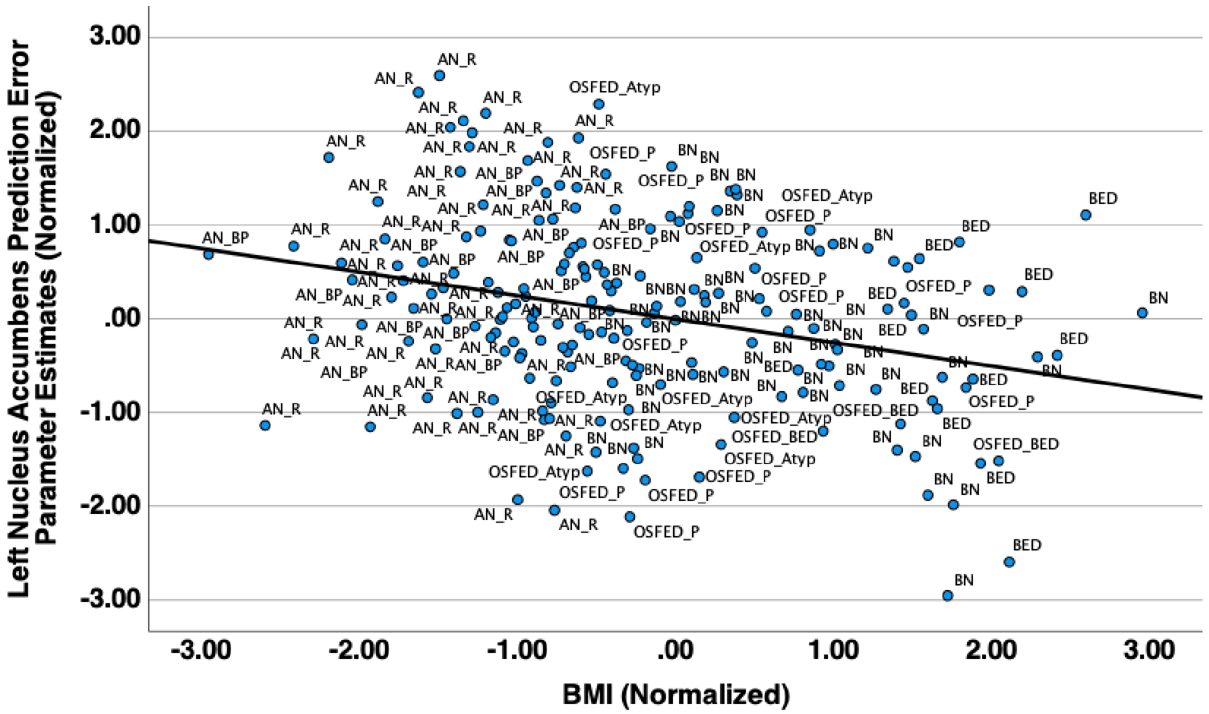

© 2021 Frank GKW et al. *JAMA Psychiatry*.

eFigure 4. Effective Connectivity Graphs for Anorexia and Bulimia Nervosa

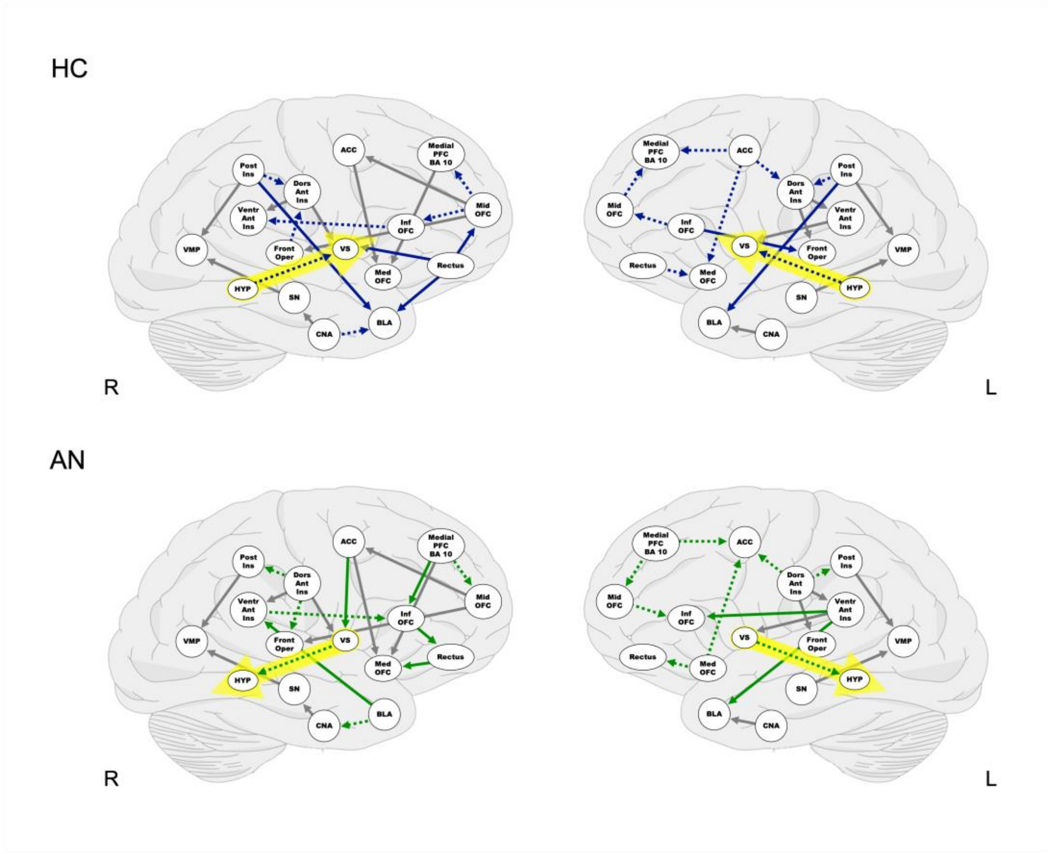

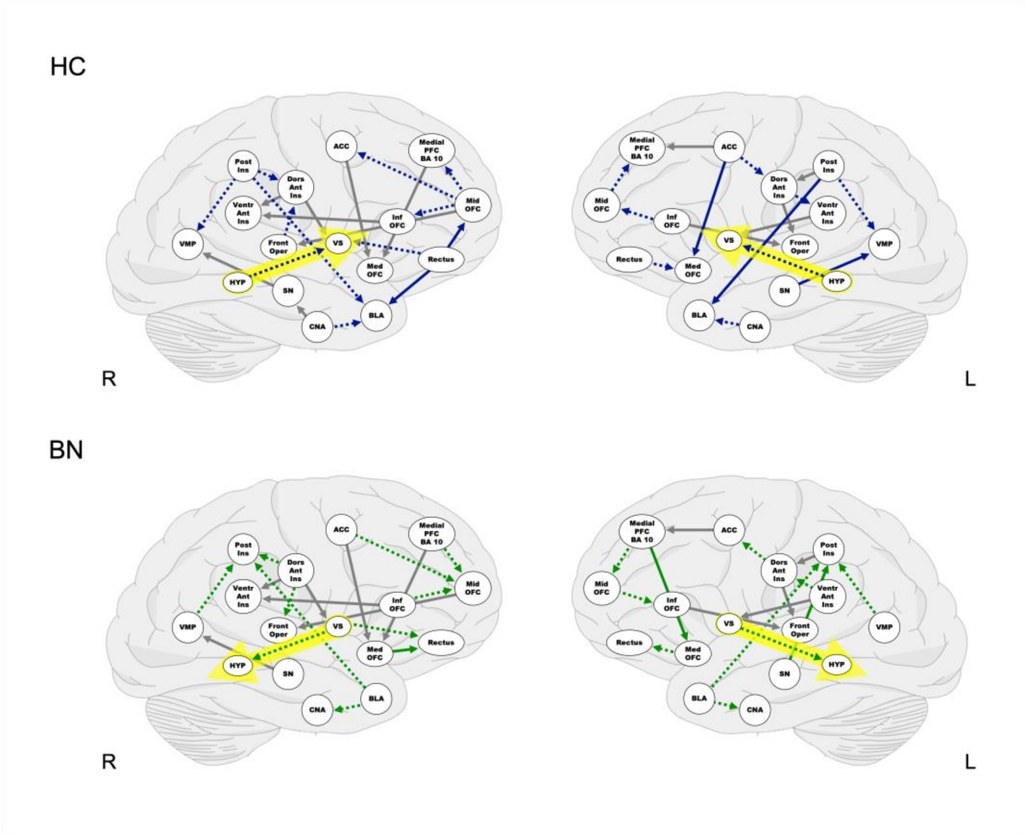

© 2021 Frank GKW et al. *JAMA Psychiatry*.

eFigure 5. Group Comparison Figure for Insula and Ventral Striatal Prediction Error Values (Normalized)

Insula

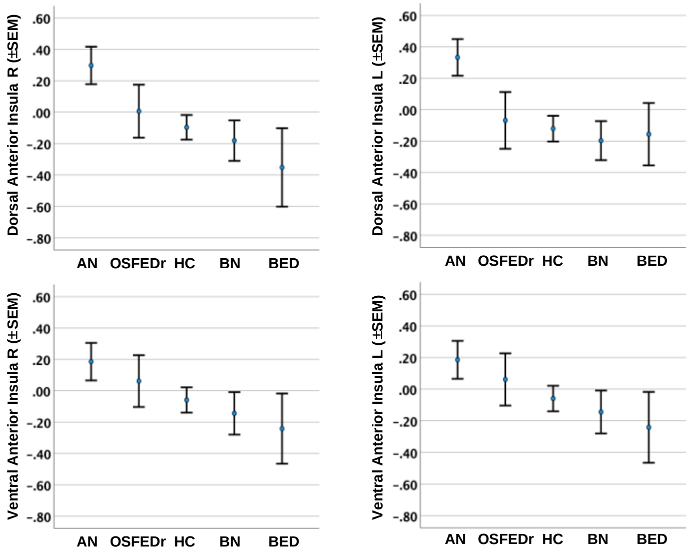

Basal Ganglia

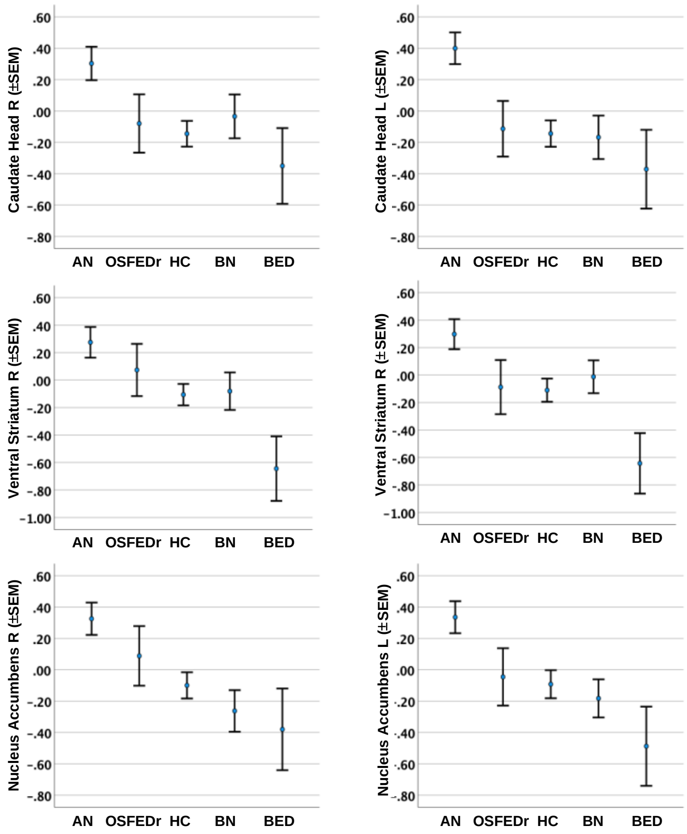

© 2021 Frank GKW et al. *JAMA Psychiatry*.

eMethods 5. Exploratory Correlation Analysis, Combined Sample

We conducted exploratory analyses using the combined study sample (n=317) and calculated correlation analyses for brain response with BMI, EDI-Bulimia, Weekly Binge Eating Frequency and Trait Anxiety. Theses analyses indicated inverse relationships between prediction error response and BMI in bilateral dorsal anterior insula (R: r=-.187, p=.003, CI95%=-.298 to CI95%=-.064; L: r=-.196, p<.001, CI95%=-.305 to CI95%=-.075), left ventral anterior insula (r=-.177, p=.002, CI95%=-.293 to CI95%=-.045), bilateral caudate head (R: r=-.209, p<.001, CI95%=-.323 to CI95%=-.094; L: r=-.260, p<.001, CI95%=-.361 to CI95%=-.156), bilateral ventral striatum (R: r=-.219, p<.001, CI95%=-.326 to CI95%=-.102; L: r=-.218, p<.001, CI95%=-.323 to CI95%=-.107), and bilateral nucleus accumbens (R: r=-.224, p<.001, CI95%=-.335 to CI95%=-.117; L: r=-.244, p<.001, CI95%=-.346 to CI95%=-.141). Fisher Z transformation to compare correlations between the Eating Disorder and Eating Disorder+Healthy Control groups did not show significant group differences. Furthermore, correlations between EDI-Bulimia, Weekly Binge Eating Frequency and Trait Anxiety and prediction error brain response were not significant in the combined sample.

© 2021 Frank GKW et al. *JAMA Psychiatry*.

eReferences

1. O'Doherty JP, Dayan P, Friston K, Critchley H, Dolan RJ. Temporal difference models and reward-related learning in the human brain. *Neuron.* 2003;38(2):329-337.

2. Frank G, Kaye W, Carter C, et al. The evaluation of brain activity in response to taste stimuli--a pilot study and method for central taste activation as assessed by event related fMRI. *J Neurosci Methods.* 2003;131(1-2):99-105.

3. Frank GK, Reynolds JR, Shott ME, O'Reilly RC. Altered temporal difference learning in bulimia nervosa. *Biol Psychiatry.* 2011;70(8):728-735.

4. Jensen J, Smith AJ, Willeit M, et al. Separate brain regions code for salience vs. valence during reward prediction in humans. *Hum Brain Mapp.* 2007;28(4):294-302.

5. Shott ME, Pryor TL, Yang TT, Frank GKW. Greater insula white matter fiber connectivity in women recovered from anorexia nervosa. *Neuropsychopharmacology.* 2015(February):1-10.

6. Frank GK, Reynolds JR, Shott ME, et al. Anorexia nervosa and obesity are associated with opposite brain reward response. *Neuropsychopharmacology.* 2012;37(9):2031-2046.

7. Seymour B, O'Doherty JP, Koltzenburg M, et al. Opponent appetitive-aversive neural processes underlie predictive learning of pain relief. *Nat Neurosci.* 2005;8(9):1234-1240.

8. Robinson OJ, Overstreet C, Charney DR, Vytal K, Grillon C. Stress increases aversive prediction error signal in the ventral striatum. *Proc Natl Acad Sci U S A.* 2013;110(10):4129-4133.

9. Seymour B, protectO'Doherty JP, Dayan P, et al. Temporal difference models describe higher order learning in humans. *Nature.* 2004;429:664-667.

10. O'Doherty JP, Buchanan TW, Seymour B, Dolan RJ. Predictive neural coding of reward preference involves dissociable responses in human ventral midbrain and ventral striatum. *Neuron.* 2006;49(1):157-166.

11. DeGuzman M, Shott ME, Yang TT, Riederer J, Frank GKW. Association of elevated reward prediction error response with weight gain in adolescent anorexia nervosa. *Am J Psychiatry.* 2017;174(6):557-565.

© 2021 Frank GKW et al. *JAMA Psychiatry*.

12. Fouragnan E, Queirazza F, Retzler C, Mullinger KJ, Philiastides MG. Spatiotemporal neural characterization of prediction error valence and surprise during reward learning in humans. *Sci Rep.* 2017;7(1):4762.

13. D'Ardenne K, Lohrenz T, Bartley KA, Montague PR. Computational heterogeneity in the human mesencephalic dopamine system. *Cogn Affect Behav Neurosci.* 2013;13(4):747-756.

14. Diederen KMJ, Fletcher PC. Dopamine, Prediction Error and Beyond. *Neuroscientist.* 2021;27(1):30-46.

15. Schultz W. Recent advances in understanding the role of phasic dopamine activity. *F1000Res.* 2019;8.

16. Fischer AG, Ullsperger M. An Update on the Role of Serotonin and its Interplay with Dopamine for Reward. *Front Hum Neurosci.* 2017;11:484.

17. Morita K, Kawaguchi Y. A dual role hypothesis of the cortico-basal-ganglia pathways: opponency and temporal difference through dopamine and adenosine. *Front Neural Circuits.* 2018;12:111.

18. Verhagen LA, Luijendijk MC, Korte-Bouws GA, Korte SM, Adan RA. Dopamine and serotonin release in the nucleus accumbens during starvation-induced hyperactivity. *Eur Neuropsychopharmacol.* 2009;19(5):309-316.

19. Frank GK, Shott ME, Riederer J, Pryor T. Altered structural and effective connectivity in anorexia and bulimia nervosa in circuits that regulate energy and reward Homeostasis. *Translational Psychiatry.* 2016; 6(11):e932.

20. Ramsey JD, Hanson SJ, Hanson C, Halchenko YO, Poldrack RA, Glymour C. Six problems for causal inference from fMRI. *Neuroimage.* 2010;49(2):1545-1558.

21. Ramsey JD, Hanson SJ, Glymour C. Multi-subject search correctly identifies causal connections and most causal directions in the DCM models of the Smith et al. simulation study. *Neuroimage.* 2011;58(3):838-848.

© 2021 Frank GKW et al. *JAMA Psychiatry*.
